# Atrial fibrillation worsens outcome of mitral valve repair for degenerative mitral regurgitation: Long-term follow-up of 959 patients

**DOI:** 10.1016/j.xjon.2026.101707

**Published:** 2026-03-02

**Authors:** Satoshi Kainuma, Naonori Kawamoto, Takashi Kakuta, Kizuku Yamashita, Kota Suzuki, Ayumi Ikuta, Rieko Kutsuzawa, Yuki Tadokoro, Shinichi Kurashima, Yuki Irie, Kenji Moriuchi, Masashi Amano, Atsushi Okada, Makoto Amaki, Hideaki Kanzaki, Takeshi Kitai, Chisato Izumi, Kazuhiro Yamamoto, Katsuhiro Omae, Satsuki Fukushima

**Affiliations:** a Department of Cardiac Surgery, National Cerebral and Cardiovascular Center, Osaka, Japan; b Department of Cardiovascular Medicine, National Cerebral and Cardiovascular Center, Osaka, Japan; c Department of Data Science, National Cerebral and Cardiovascular Center, Osaka, Japan

**Keywords:** mitral repair, degenerative mitral regurgitation, atrial fibrillation

## Abstract

**Background:**

The prognostic significance of atrial fibrillation (AF) in patients with degenerative mitral regurgitation (MR) undergoing mitral valve (MV) repair remains controversial. The association between preexisting AF and long-term clinical and echocardiographic outcomes following mitral repair was analyzed.

**Methods:**

This retrospective study included 959 patients (mean age, 60 ± 12 years) who underwent MV repair for degenerative MR between 2001 and 2022. Patients were stratified by preoperative rhythm: 670 in sinus rhythm and 289 with AF. Serial echocardiographic changes were assessed using linear mixed models. The mean follow-up was 8.6 ± 5.4 years (8219 patient-years).

**Results:**

Compared to patients with sinus rhythm, those with AF were older, more symptomatic, and exhibited greater cardiac remodeling, prompting higher rates of concomitant tricuspid annuloplasty (45% vs 5.7%) and ablation procedures (91% vs 0%) (*P* < .05 for all). At 10 years, AF patients had lower overall survival (89% vs 96%; *P* = .003) and freedom from composite adverse events—mitral reintervention, heart failure, stroke, catheter ablation, or pacemaker implantation (56% vs 80%; *P* < .001). Propensity score matching (n = 239 pairs) for 15 clinical covariates confirmed AF as an independent predictor of adverse events (adjusted hazard ratio, 1.8; 95% confidence interval, 1.3-2.5; *P* < .001). Additionally, AF patients demonstrated less improvement in left atrial size, tricuspid regurgitation (TR) pressure gradient, and TR severity up to 10 years (group effect *P* < .001).

**Conclusions:**

These findings indicate that AF identifies a higher-risk phenotype characterized by advanced atrial disease and persistent hemodynamic burden following MV repair. Future studies are needed to determine whether intervention before the development of advanced atrial remodeling or AF can improve long-term outcomes.

## Graphical Abstract

**Figure:**
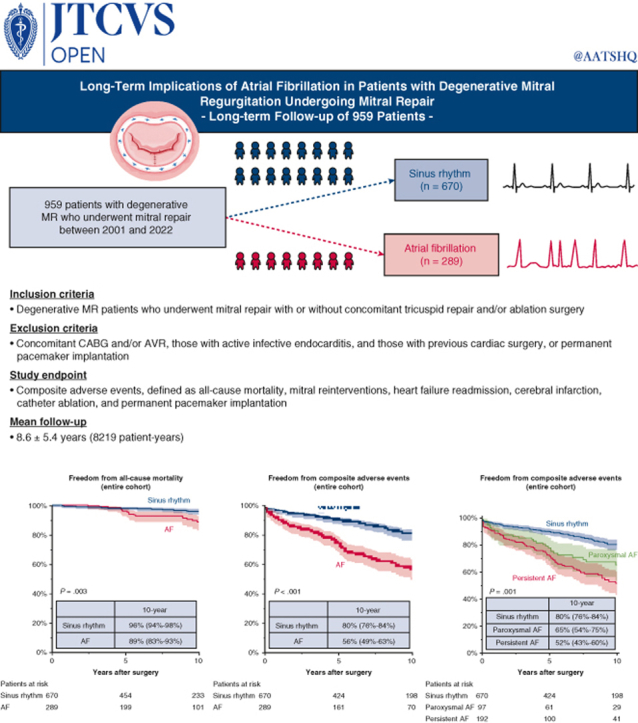

**Figure:**
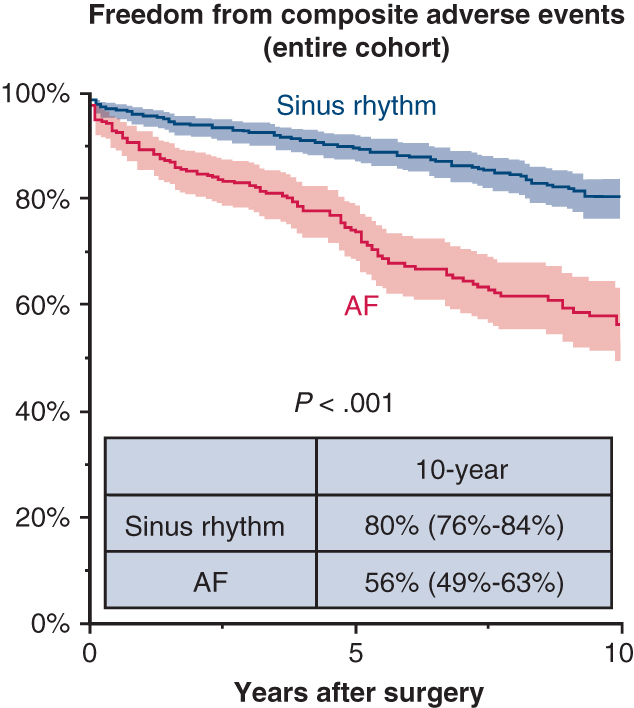
Freedom from composite adverse events between patients in sinus rhythm and those with atrial fibrillation. Shaded area represents 95% confidence interval.

**Central Message**

Despite aggressive concomitant rhythm and valve interventions, preexisting atrial fibrillation was associated with worse long-term outcomes, attenuated left atrial reverse remodeling, and unfavorable hemodynamics.

**Perspective**

In patients with degenerative mitral regurgitation undergoing mitral valve repair, preexisting atrial fibrillation (AF) reflects advanced atrial remodeling and portends worse long-term outcomes despite aggressive concomitant rhythm and valve interventions. Recognition of AF should prompt comprehensive evaluation and guideline-informed discussion regarding operative timing, rhythm management, and long-term surveillance.

Degenerative mitral regurgitation (MR) is the most prevalent form of valvular heart disease and a leading indication for mitral valve (MV) repair worldwide.^1^ MV repair is the gold standard for severe degenerative MR owing to low operative mortality, superior long-term survival, reduced thromboembolic risk, and excellent durability, often restoring life expectancy to that of the general population across age groups.^1^, ^2^, ^3^, ^4^, ^5^, ^6^ Despite these proven benefits, the optimal timing of surgical intervention remains a topic of debate.

Atrial fibrillation (AF) frequently occurs in chronic degenerative MR as a consequence of left atrial (LA) enlargement and fibrosis induced by volume overload.^7^, ^8^, ^9^, ^10^ Several studies have shown that AF is associated with increased mortality in degenerative MR, independent of baseline characteristics.^7^^,^^9^ In the largest international registry (Mitral Regurgitation International Database; MIDA), both paroxysmal and persistent AF were associated with significantly lower survival compared with sinus rhythm both before and after surgery.^9^ Current guidelines acknowledge the adverse prognostic significance of AF and recommend considering earlier surgery and concomitant AF procedures in suitable candidates^5^^,^^11^^,^^12^; however, prior registries contained relatively few patients undergoing ablation, limiting dedicated analyses.^9^ Clarifying the association of preexisting AF with long-term outcomes following MV repair can refine the timing and strategy of intervention. This study analyzed the impact of baseline AF on adverse events after MV repair for degenerative MR.

## Methods

The institutional surgical database contained a consecutive series of 1045 patients with degenerative MR who underwent MV repair with or without concomitant tricuspid valve repair and/or ablation surgery at the National Cerebral and Cardiovascular Center hospital between 2001 and 2022. We excluded patients who underwent concomitant coronary artery bypass grafting (n = 60) or aortic valve replacement (n = 4), those with active infective endocarditis (n = 9), and those with previous cardiac surgery (n = 10) or a permanent pacemaker implantation (n = 3). Finally, 959 patients (mean age, 60 ± 12 years) were included in this study.

For the specific purpose of this study, preoperative cardiac rhythm had to be ascertained by electrocardiography, and those patients were classified according to baseline cardiac rhythm: 670 with sinus rhythm and 289 with AF (97 paroxysmal, 192 persistent). The Institutional Review Board of the National Cerebral and Cardiovascular Center approved the data collection, analysis, and reporting (reference M30-026; approved March 25, 2022). All patients enrolled in the study provided written informed consent to have their information released and for the publication of study data.

### Surgical Indications and Techniques

The surgical indications for MV repair, operative approaches, and repair techniques have been reported previously.^13^ At our institution, surgical ablation using cryothermia for the treatment of AF has been performed since 2001, as described previously.^14^ For left-sided ablation, a standard right-sided left atriotomy was performed, and box lesions encircling the bilateral pulmonary veins, the LA appendage, and the mitral annulus were created to connect the incision line. Since 2019, the coronary sinus also has been ablated via the oblique sinus. For right-sided ablation, the right atrium was incised longitudinally, and lesions were created at the fossa ovalis, tricuspid annulus, and the orifice of the inferior vena cava to connect the incision line. Exclusion of the LA appendage was achieved either by oversewing the orifice from the endocardial side or by external clipping using an AtriClip device (AtriCure). Biatrial ablation (cryo-maze) was performed whenever feasible; however, the indications for surgical ablation and LA appendage management, as well as the lesion set, were determined based on a comprehensive risk–benefit assessment, including the degree of AF progression (ie, AF type and duration, LA volume, and F-wave voltage), thromboembolic risk, severity of AF-related symptoms, and patient compliance with anticoagulant therapy.^15^

### Clinical Follow-up Examinations

The primary study endpoint was composite adverse events, defined as all-cause mortality, mitral reinterventions, heart failure readmission, cerebral infarction, catheter ablation, and permanent pacemaker implantation. To evaluate longitudinal changes in left ventricular (LV) function parameters, LA dimension, tricuspid regurgitation (TR) pressure gradient, and MR severity, transthoracic 2-dimensional and Doppler echocardiography were performed before mitral surgery, at 1 week after surgery, and annually thereafter whenever possible. MR was defined as grade 0, no MR; grade 1, trivial; grade 2, mild; grade 3, moderate; or grade 4, severe. Clinical outcomes were assessed throughout follow-up. In contrast, serial echocardiographic analyses were censored at the time of mitral reintervention to avoid mixing post-reintervention physiology with postrepair remodeling; a last-observation-carried-forward approach was not used.

### Statistical Analysis

Continuous variables are summarized as mean ± standard deviation and were compared using the unpaired *t* test or Mann-Whitney *U* test. Categorical variables are summarized as frequency with proportion and were compared using the χ^2^ test or Fisher exact test, as appropriate. Serial echocardiographic changes were analyzed using linear mixed-effects models with group (AF vs sinus rhythm), time (preoperative, immediately after surgery, and 1, 3, 5, 7, and 10 years after surgery), and their interaction (group × time) as fixed effects. A random intercept for each subject was included to account for within-subject correlation, and an unstructured covariance matrix was assumed for the repeated measures. Time-to-event distributions were estimated by the Kaplan-Meier method and compared using the a log-rank test.

Fifteen clinically relevant baseline and surgical covariates—age, sex, body surface area, symptomatic, hypertension, hyperlipidemia, diabetes, chronic obstructive pulmonary disease, serum creatinine level, LV end-diastolic dimension (LVEDD), LV end-systolic dimension (LVESD), LV ejection fraction (LVEF), TR severity, location of leaflet prolapse, and surgical MV repair approaches—were adjusted using a propensity score matching technique with a 1:1 nearest-available matching algorithm within a caliper width of 0.2 without replacement. Propensity scores were calculated using multivariable logistic regression including the covariates listed above. Covariate balance before and after matching was assessed using standardized mean difference (SMD); an SMD <0.25 (25%) was considered to indicate a negligible imbalance between groups.

The association between preoperative AF and composite adverse events was evaluated using Cox proportional hazards models. Variables with *P* < .05 in univariable analysis and those considered clinically relevant were included in multivariable models. Results are presented as hazard ratio (HR) with 95% confidence interval (CI). All *P* values are 2-sided, and *P* < .05 was considered to indicate statistical significance. Because this was an exploratory observational study, no adjustment for multiplicity was performed. Statistical analyses were performed using JMP 7.0 (SAS Institute) and R version 3.4.3 (R Foundation for Statistical Computing).

## Results

### Preoperative Patient Characteristics

Patient characteristics and preoperative echocardiography are listed in Table 1. In an unmatched cohort, there were no intergroup differences in sex, body surface area, prevalence of hypertension, hyperlipidemia, or diabetes; however AF patients were older (mean age 66 ± 11 years vs 58 ± 13 years), were more likely to be symptomatic (87% vs 72%) and to present with chronic obstructive pulmonary disease (24% vs 11%), and had higher serum creatinine levels (mean, 0.9 ± 0.6 mg/dL vs 0.8 ± 0.2 mg/dL). On echocardiography, those patients showed larger LVESD (36 ± 5.9 mm vs 35 ± 5.3 mm), lower LVEF (60 ± 8.3% vs 63 ± 6.9%), larger LA dimension (53 ± 8.9 mm vs 46 ± 7.1 mm), and higher prevalences of TR severity ≥moderate (22% vs 5.2%) and anterior or bileaflet prolapse (46% vs 35%) (*P* < .05 for all). When patients with baseline AF were stratified according to AF type (ie, paroxysmal vs persistent), those with persistent AF exhibited larger LA size (55 ± 8.9 mm vs 48 ± 6.8 mm; *P* < .001) and a higher prevalence of TR severity ≥moderate (28% vs 10%; *P* < .001).

**Table 1:** Patient demographics before and after propensity score matching

Variables	Unmatched				Matched			
	Sinus rhythm (N = 670)	AF (N = 289)	*P* value	SMD	Sinus rhythm (N = 239)	AF (N = 239)	*P* value	SMD
Clinical variables								
Age, y, mean ± SD	58 ± 13	66 ± 11	<.001	0.664	64 ± 11	64 ± 10	.950	0.006
Male sex, n (%)	407 (61)	188 (65)	.206	0.083	166 (69)	154 (64)	.243	0.106
Body surface area, m^2^, mean ± SD	1.65 ± 0.20	1.62 ± 0.19	.060	0.154	1.64 ± 0.20	1.63 ± 0.20	.630	0.050
Symptomatic, n (%)	484 (72)	250 (87)	<.001	0.378	206 (86)	203 (85)	.696	0.028
Hypertension, n (%)	271 (40)	130 (45)	.192	0.101	110 (46)	104 (44)	.581	0.040
Hyperlipidemia, n (%)	159 (24)	66 (23)	.764	0.024	62 (26)	58 (24)	.673	0.046
Diabetes, n (%)	35 (5.2)	24 (8.3)	.076	0.124	21 (8.8)	17 (7.1)	.499	0.063
COPD, n (%)	72 (11)	68 (24)	<.001	0.347	40 (17)	44 (18)	.631	0.026
Creatinine, mg/dL, mean ± SD	0.8 ± 0.2	0.9 ± 0.6	<.001	0.289	0.9 ± 0.2	0.9 ± 0.2	.767	0.044
Echocardiographic data								
LVEDD, mm, mean ± SD	58 ± 6.0	57 ± 6.5	.376	0.160	57 ± 6.5	57 ± 6.4	.763	0.031
LVESD, mm, mean ± SD	35 ± 5.3	36 ± 5.9	.019	0.178	36 ± 5.9	36 ± 5.7	.733	0.034
LVEF, %, mean ± SD	63 ± 6.9	60 ± 8.3	<.001	0.377	61 ± 7.7	61 ± 7.8	.879	0.013
LA dimension, mm, mean ± SD	46 ± 7.1	53 ± 8.9	<.001	0.870	47 ± 6.9	52 ± 8.7	<.001	0.637
TR grade ≥moderate, n (%)	35 (5.2)	63 (22)	<.001	0.506	24 (10)	32 (13)	.255	0.094
Leaflet involvement, n (%)								
Posterior only	435 (65)	156 (54)	.002	0.226	138 (58)	136 (57)	.853	0.020
Anterior or bileaflet	235 (35)	133 (46)			101 (42)	103 (43)		

*SMD*, Standardized mean difference; *SD*, standard deviation; *COPD*, chronic obstructive pulmonary disease; *LVEDD*, left ventricular end-diastolic dimension; *LVESD*, left ventricular end-systolic dimension; *LVEF*, left ventricular ejection fraction; *LA*, left atrial; *TR*, tricuspid regurgitation.

**Figure E1:**
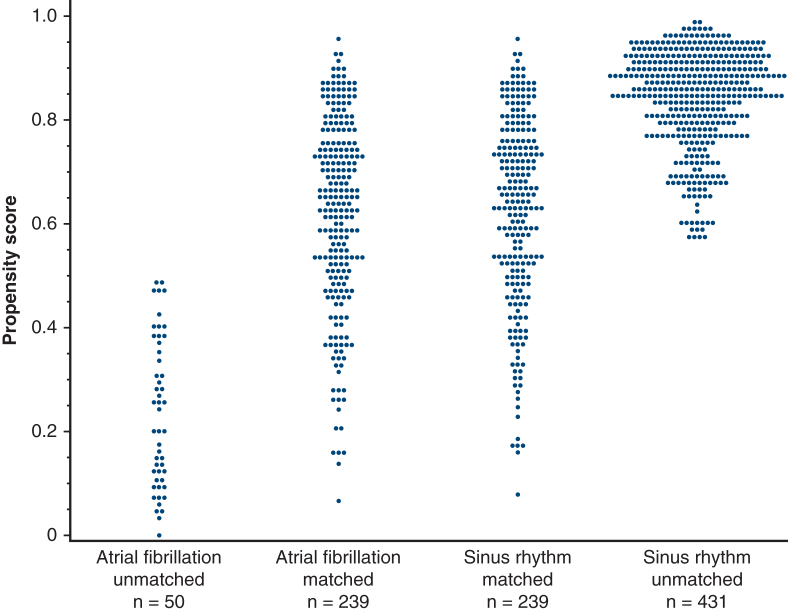
Jitter plot indicating the distribution of propensity scores for matched and unmatched patients with atrial fibrillation (*AF*) and sinus rhythm.

One-to-one matched analysis using the estimated propensity score based on 15 covariates resulted in 239 well-matched patient pairs (Figure E1). Model discrimination was assessed using C-statistics (C = 0.775). After adjustment, no intergroup differences were found in any of the baseline covariates except LA dimension, with an SMD <0.15 (15%) for each covariate.

### Operative Data and Early Outcomes

Surgical procedures and early outcomes are summarized in Table 2. AF patients were more likely to receive mitral repair via conventional sternotomy (57% vs 39%) and to undergo concomitant tricuspid annuloplasty (45% vs 5.7%), surgical ablation (91% vs 0%), and LA appendage closure (62% vs 0.6%). Among 264 patients with AF who underwent surgical ablation, biatrial ablation (cryo-maze) was performed in the majority of cases (n = 239, 91%). Among patients undergoing LA appendage exclusion, the most frequently used technique was epicardial clip occlusion (45%), followed by endocardial suturing (internal ligation; 43%), external ligation (10%), and other techniques (1.1%), with no significant differences between the groups (*P* = .782). A complete mitral ring was implanted more frequently in AF patients (34% vs 17%), with a comparable annuloplasty ring size. Mitral repair using the resection and suture technique was slightly less frequent in AF patients. Consequently, the AF group had longer operation times, more frequent use of transfusion, and longer stays in the intensive care unit and hospital, but there was no difference in the rate of in-hospital mortality (0% vs 0.1%). In a propensity score–matched cohort, substantially similar results were observed for operative data and early outcomes.

**Table 2:** Surgical data and early outcomes

Variables	Unmatched			Matched		
	Sinus rhythm (N = 670)	AF (N = 289)	*P* value	Sinus rhythm (N = 239)	AF (N = 239)	*P* value
Approach, n (%)						
Sternotomy	263 (39)	166 (57)	<.001	140 (59)	132 (55)	.460
Right thoracotomy	407 (61)	123 (43)		99 (41)	107 (45)	
Concomitant procedures, n (%)						
Tricuspid annuloplasty	38 (5.7)	129 (45)	<.001	26 (11)	91 (38)	<.001
Surgical ablation	0 (0)	264 (91)	<.001	0 (0)	223 (93)	<.001
Biatrial ablation	0 (0)	239 (83)		0 (0)	200 (84)	
Left-sided ablation	0 (0)	25 (8.7)		0 (0)	23 (9.6)	
Left atrial appendage closure	4 (0.6)	179 (62)	<.001	3 (1.3)	145 (61)	<.001
Mitral annuloplasty ring, n (%)						
Partial	558 (83)	192 (66)	<.001	196 (82)	165 (69)	<.001
Complete	112 (17)	97 (34)		43 (18)	74 (31)	
Annuloplasty ring size, mean ± SD	30 ± 2.5	30 ± 2.5	.538	29 ± 2.4	30 ± 2.6	.015
Repair technique, n (%)						
Resection and suture	366 (55)	136 (47)	.046	127 (53)	117 (49)	.327
Chordal replacement	225 (34)	117 (40)		84 (35)	98 (41)	
Both	59 (8.8)	21 (7.3)		23 (9.6)	16 (6.7)	
Others	20 (3.0)	15 (5.2)		5 (2.1)	8 (3.4)	
Early outcomes						
Operation time, min, mean ± SD	229 ± 64	275 ± 69	<.001	232 ± 63	272 ± 67	<.001
CPB time, min, mean ± SD	117 ± 40	151 ± 46	<.001	116 ± 39	149 ± 45	<.001
Cardiac arrest time, min, mean ± SD	79 ± 28	109 ± 33	<.001	78 ± 27	108 ± 33	<.001
Transfusion, n (%)	214 (32)	153 (53)	<.001	103 (43)	115 (48)	.289
ICU stay, d, mean ± SD	1.7 ± 1.0	2.0 ± 1.3	<.001	1.8 ± 1.0	1.9 ± 1.1	.110
Hospital stay, d, mean ± SD	11 ± 5.4	13 ± 7.3	<.001	12 ± 5.7	13 ± 7.1	.169
In-hospital mortality, n (%)	1 (0.1)	0 (0)	1.000	1 (0.4)	0 (0)	1.000

*SD*, Standard deviation; *CPB*, cardiopulmonary bypass time; *ICU*, intensive care unit.

### Long-Term Outcomes

Our cohort’s long-term outcomes are summarized in Table 3. Clinical follow-up examinations were completed with a mean follow-up of 8.6 ± 5.4 years (8219 patient-years). During follow-up, there were 67 deaths, 69 mitral reinterventions, 49 heart failure readmissions, 60 strokes, 40 catheter ablations, and 20 pacemaker implantations. Causes of death included malignancy (n = 14; 21%), unknown (n = 11; 16%), pneumonia (n = 9; 13%), cerebrovascular events (n = 7; 10%), heart failure (n = 7; 10%), senile deterioration (n = 7; 10%), sudden death (n = 5; 7.5%), gastrointestinal bleeding (n = 2; 3.0%), renal failure (n = 2; 3.0%), others (n = 2; 3.0%), and ventricular arrhythmia (n = 1; 1.5%). A total of 69 patients had an MV-related reoperation, including 37 for recurrent MR, 16 for hemolysis with MR, 7 for endocarditis, 6 for mitral stenosis due to pannus, 2 for LA thrombus, and 1 for systolic motion of the MV anterior leaflet. The MV was re-repaired in 15 patients and replaced in 52, and thrombectomy was performed in 2 patients.

**Table 3:** Long-term clinical and rhythm outcomes

Outcome	Unmatched			Matched		
	Sinus rhythm (n = 670)	AF (n = 289)	*P* value	Sinus rhythm (n = 239)	AF (n = 239)	*P* value
Long-term outcomes						
Follow-up period, y, mean ± SD	8.5 ± 5.4	8.7 ± 5.5	.613	9.0 ± 5.4	8.8 ± 5.7	.776
All-cause death, n (%)	35 (5.2)	32 (11)	.002	20 (8.4)	25 (10)	.433
Mitral reoperation, n (%)	45 (6.7)	24 (8.3)	.388	15 (6.3)	20 (8.4)	.379
Heart failure readmission, n (%)	21 (3.1)	28 (9.7)	<.001	11 (4.6)	23 (9.6)	.031
Cerebral infarction, n (%)	30 (4.5)	30 (10)	<.001	14 (5.9)	21 (8.8)	.218
Catheter ablation, n (%)	20 (3.0)	20 (6.9)	.007	7 (2.9)	17 (7.1)	.034
Pacemaker implant, n (%)	7 (1.0)	13 (4.5)	.001	3 (1.3)	9 (3.8)	.073
Rhythm outcomes, n (%)						
1-y follow-up	n = 657	n = 288		n = 232	n = 238	
Sinus rhythm	653 (99)	250 (87)	<.001	230 (99)	213 (90)	<.001
AF	1 (0.2)	30 (10)		1 (0.4)	19 (8.0)	
Pacemaker rhythm	3 (0.5)	8 (2.8)		1 (0.4)	6 (2.5)	
3-y follow-up	n = 504	n = 247		n = 177	n = 201	
Sinus rhythm	498 (99)	202 (82)	<.001	175 (99)	170 (85)	<.001
AF	4 (0.8)	35 (14)		1 (0.6)	24 (12)	
Pacemaker rhythm	2 (0.4)	10 (4.1)		1 (0.6)	7 (3.5)	
5-y follow-up	n = 377	n = 189		n = 133	n = 155	
Sinus rhythm	368 (98)	154 (81)	<.001	129 (97)	132 (85)	.001
AF	7 (1.9)	25 (13)		3 (2.3)	16 (10)	
Pacemaker rhythm	2 (0.5)	10 (5.3)		1 (0.8)	7 (4.5)	
7-y follow-up	n = 276	n = 146		n = 96	n = 121	
Sinus rhythm	266 (96)	100 (68)	<.001	91 (95)	89 (74)	<.001
AF	7 (2.5)	36 (25)		3 (3.1)	26 (21)	
Pacemaker rhythm	3 (1.1)	10 (6.9)		2 (2.1)	6 (5.0)	
10-y follow-up	n = 164	n = 98		n = 58	n = 84	
Sinus rhythm	150 (91)	62 (63)	<.001	55 (95)	56 (67)	<.001
AF	11 (6.7)	28 (29)		2 (3.5)	23 (27)	
Pacemaker rhythm	3 (1.8)	8 (8.2)		1 (1.7)	5 (6.0)	

*AF*, Atrial fibrillation.

**Figure 1:**
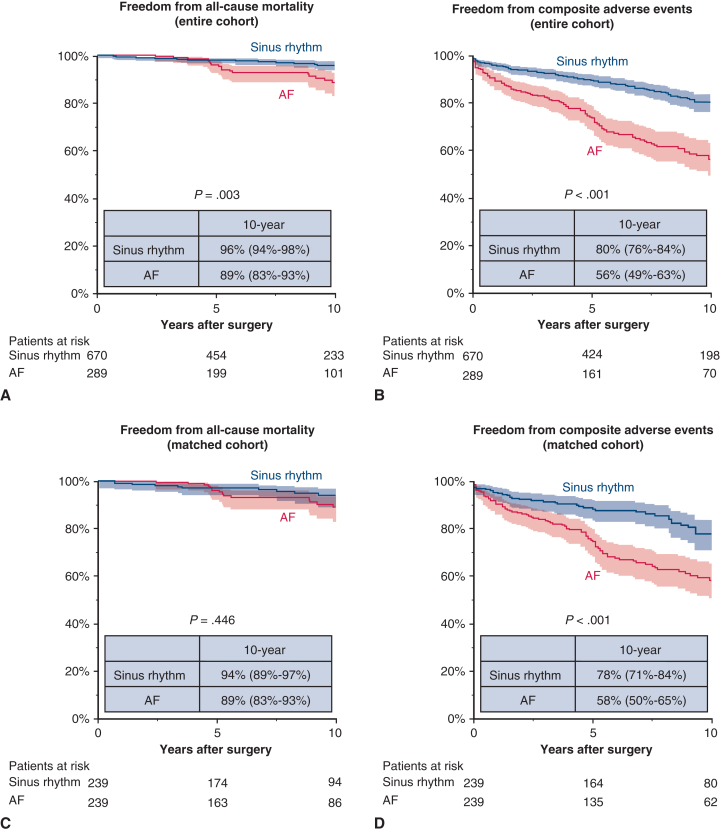
Kaplan-Meier estimates of freedom from all-cause mortality and composite adverse events. A and B, Results for the unmatched cohort. C and D, Results for the propensity score–matched cohort. Shaded areas represent 95% confidence intervals. Numbers at risk are shown below each panel. *AF*, Atrial fibrillation.

While there was no between-group difference in the rate of mitral reintervention (8.3% for AF vs sinus rhythm 6.7%), AF patients were more likely to experience all-cause death (11% vs 5.2%; *P* = .002), heart failure readmission (9.7% vs 3.1%; *P* < .001), cerebral infarction (10% vs 4.5%; *P* < .001), catheter ablation (6.9% vs 3.0%; *P* = .007), and permanent pacemaker implantation (4.5 vs 1.0%; *P* = .001) (Table 3). Consequently, AF patients showed lower rates of 10-year survival (89% vs 96%; *P* = .003) and freedom from composite events (56% vs 80%; *P* < .001) (Figure 1, *A* and *B*).

**Figure E2:**
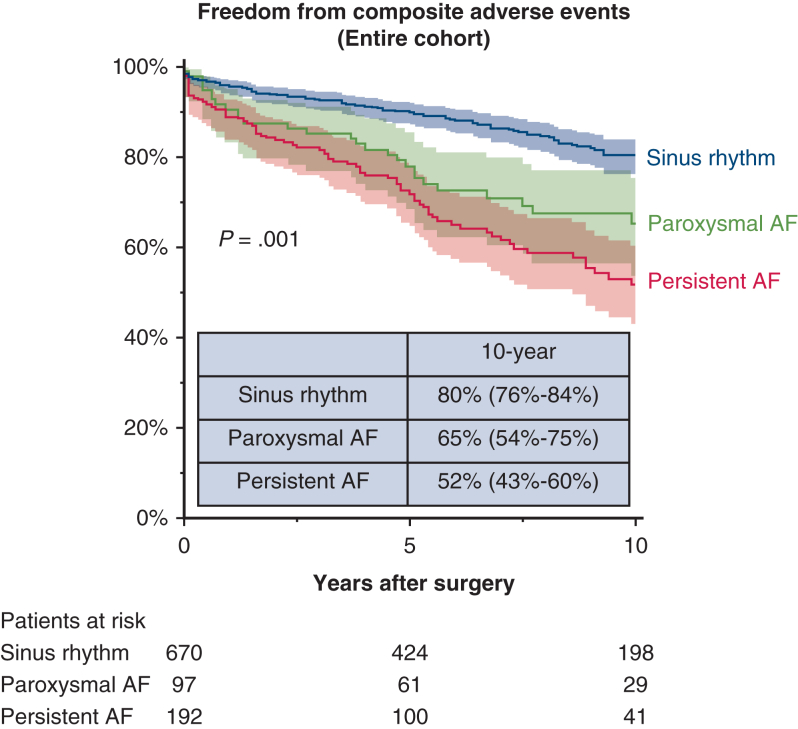
Kaplan-Meier estimates of composite adverse events among patients with sinus rhythm, paroxysmal atrial fibrillation (*AF*), and persistent AF in the unmatched cohort. Shaded areas represent 95% confidence intervals, and numbers at risk are shown below the plot.

In a propensity score–matched cohort, AF patients still showed higher rates of heart failure readmission (9.6% vs 4.6%; *P* = .031) and catheter ablation (7.1% vs 2.9%; *P* = .034) (Table 3). Although there was no significant between-group difference in the survival rate (89% vs 94%; *P* = .446), AF patients had a lower 10-year freedom from composite events (58% vs 78%; *P* < .001) (Figure 1, *C* and *D*). When patients with baseline AF were stratified by AF type, those with persistent AF had lower freedom from composite adverse events than those with paroxysmal AF (Figure E2).

### Long-Term Rhythm Outcomes

The prevalence of sinus rhythm among patients who were in sinus rhythm at baseline was 99% at 1 year, 99% at 3 years, 98% at 5 years, 96% at 7 years, and 91% at 10 years after surgery. There rates are significantly higher than the corresponding rates in patients with AF at baseline: 87%, 82%, 81%, 68%, and 63% (Table 3). This tendency also was observed in a matched cohort.

### Long-Term Echocardiographic Findings

**Figure 2:**
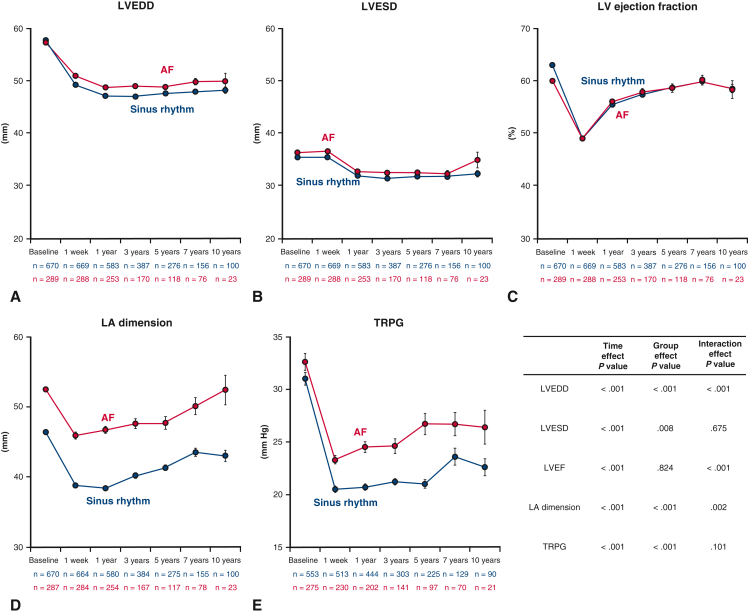
Serial echocardiographic results for left ventricular end-diastolic dimension (*LVEDD*) (A), left ventricular end-systolic dimension (*LVESD*) (B), left ventricular (*LV*) ejection fraction (C), left atrial (*LA*) dimension (D), and tricuspid regurgitation pressure gradient (*TRPG*) (E) in the 2 study groups.

**Figure 3:**
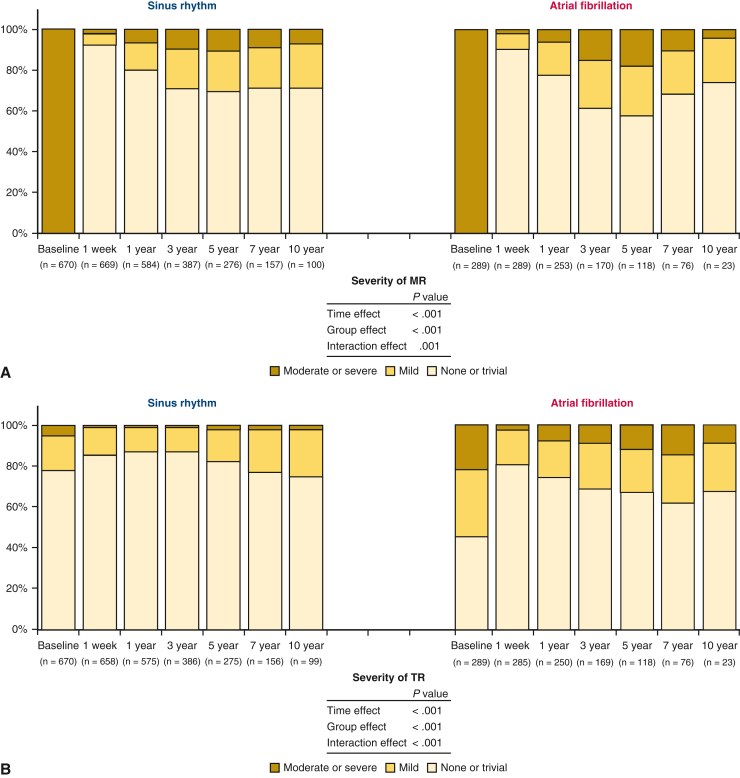
Serial echocardiographic results for mitral regurgitation (MR) (A) and tricuspid regurgitation (TR) (B) in the two study groups.

From baseline to 1 week after surgery, LVEDD was decreased, LVESD did not change, and LVEF decreased (Figure 2, *A-C*). LA dimension and TR pressure gradient were significantly decreased (Figure 2, *D* and *E*), along with significant improvements in MR grade and TR grade (Figure 3). LVEDD continued to decrease until 1 year after repair and remained stable thereafter, with modest but significantly greater improvement in patients with sinus rhythm (interaction effect *P* < .001). LVESD and LV ejection fraction improved from 1 week to 1 year, and these improvements were generally sustained thereafter (interaction effect *P* = .675 and *P* < .001 for each). Both groups showed a steady increase in LA dimensions after 1 week of repair, but patients with AF presented with larger LA dimensions at any time during follow-up, consequently yielding an enlarged LA at 10 years, almost comparable to the baseline value (interaction effect *P* < .001). Likewise, the TR pressure gradient was greater in patients with AF (group effect *P* < .001). The rate of moderate or severe MR increased after 1 week postrepair, peaking at 5 years postrepair, with a higher rate in patients with AF (interaction effect *P* = .001). Also, the rate of moderate or severe TR was greater in AF patients throughout the follow-up period (interaction effect *P* < .001).

### Association With Composite Events Following Mitral Repair

**Figure 4:**
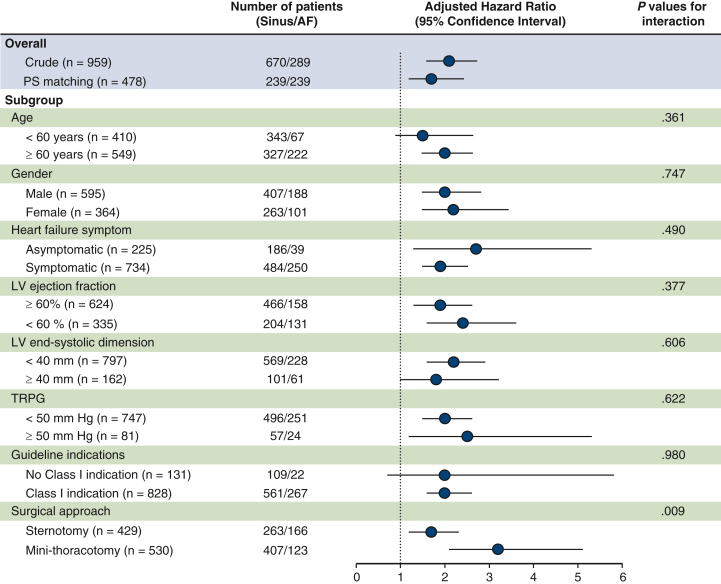
Forest plot illustrating the excess risk of composite events associated with atrial fibrillation (*AF*) overall and in subgroups of patients, according to the presence/absence of guideline-endorsed predictors of outcome in degenerative mitral regurgitation. The hazard ratio is set at 1 for sinus rhythm. The *P* value for interaction is reported on the right. The sample size of patients in each specific subgroup is reported as a number. *LV*, Left ventricular; *TRPG*, tricuspid regurgitation pressure gradient.

Among the baseline characteristics, age ≥60 years, body surface area, presence of heart failure symptoms at baseline, preoperative AF, chronic obstructive pulmonary disease, serum creatinine level, LVEF <60%, LA dimensions, TR severity ≥moderate, and isolated posterior leaflet prolapse were associated with composite adverse events on unadjusted Cox proportional hazards analysis. After adjustment for those preoperative correlates, preoperative AF was independently associated with a lower risk of composite adverse events (adjusted HR, 1.5; 95% CI, 1.1-2.0; *P* = .010) (Table E1). In a propensity score–matched cohort, preoperative AF remained an independent risk factor for composite adverse events (adjusted HR, 1.8; 95% CI, 1.3-2.5; *P* < .001) (Table E2). Moreover, a sensitivity analysis showed that the association between preoperative AF and adverse outcomes remained consistent after excluding catheter ablation from the primary composite endpoint (Table E3). The adjusted HRs for the composite adverse events in several demographic and clinical risk subgroups showed worse event-free survival for patients with AF compared to those with sinus rhythm in all subgroups (Figure 4).

## Discussion

**Figure 5:**
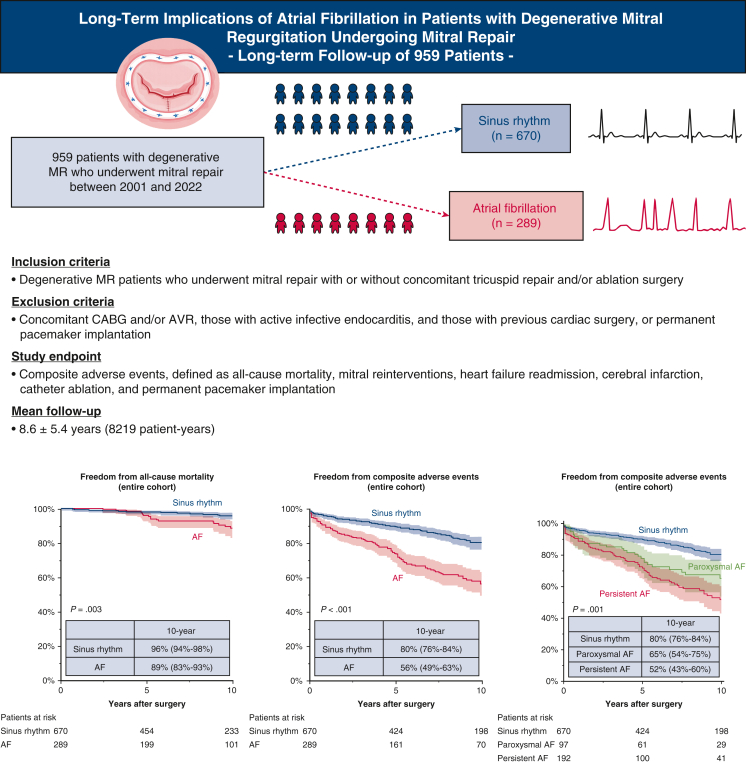
Patient enrollment, endpoints and main results including all-cause mortality (sinus vs atrial fibrillation [*AF*]), composite adverse events (sinus vs AF) and composite adverse events (sinus vs paroxysmal AF vs persistent AF) in an unmatched cohort. *MR*, Mitral regurgitation; *CABG*, coronary artery bypass grafting; *AF*, atrial fibrillation.

In this large, single-center cohort with long-term follow-up, we demonstrated that preexisting AF is strongly associated with adverse outcomes after MV repair for degenerative MR. Despite a high prevalence of concomitant ablation procedures and aggressive tricuspid repair in the AF group, these patients had significantly worse clinical outcomes in terms of higher rates of heart failure readmission, stroke, catheter ablation, and pacemaker implantation compared with those in sinus rhythm (Figure 5). On serial echocardiography, patients with AF also showed attenuated LA reverse remodeling and sustained hemodynamic stress over time even after successful MV repair. Notably, the adverse prognostic impact of AF persisted even after rigorous adjustment with propensity score matching, underscoring its independent role in influencing long-term outcomes following MV repair.

David and colleagues^16^ reported excellent long-term outcomes in a total of 1234 consecutive patients (median age 59 years; 70.4% males) undergoing mitral repair for leaflet prolapse, followed for a median of 13 years. Their study revealed 10-year cumulative incidences of 10.7% for all-cause deaths, 3.2% of mitral reoperation, 7.2% for thromboembolism of 7.2%, and 1.6% for permanent pacemaker implantation. Compared with their findings, we found higher incidences of mitral reoperation and permanent pacemaker implantation, which might have been related to differences in the location of leaflet prolapse and the incidence of AF, as well as to the prevalence of concomitant ablation procedures. Importantly, David and colleagues also found relatively high incidences of postoperative TR aggravation (20.8%), and new-onset AF (32.4%), emphasizing that postoperative arrhythmogenic remodeling may occur despite durable repair.

The observed differences in baseline characteristics between AF patients and sinus rhythm patients highlight the interplay between MR severity, atrial remodeling, and arrhythmogenesis. In our study, AF patients were older, more symptomatic, and demonstrated larger LA dimensions, more frequent TR, and worse LV function preoperatively—features that have been independently linked to adverse outcomes in prior studies.^1^^,^^7^^,^^9^^,^^10^ Our results align with several previous registry studies, including the MIDA registry, which highlighted the deleterious prognostic implications of AF in both surgical and medically managed patients with MR.^6^, ^7^, ^8^, ^9^ The MIDA registry analysis demonstrated that both paroxysmal and persistent AF at diagnosis were significantly associated with worse 10-year survival (82% for sinus, 70% for paroxysmal, and 57% for persistent AF; *P* < .001).^9^ However, consistent with the real-world clinical practice, where the opportunity to treat AF is ignored in 1 out of every 3 patients who undergo a mitral valve operation, those previous reports included relatively few patients (9.8%) undergoing concomitant ablation procedures, limiting the ability to evaluate the influence of rhythm control at the time of mitral surgery.^17^ In contrast, in our series, the majority of AF patients underwent concomitant ablation procedures and LA appendage closure, yet adverse outcomes remained more frequent. Our study adds robust evidence supporting AF as an independent adverse prognostic marker, signaling more extensive atrial remodeling, including dilation and functional impairment of atrial reservoir function—pathophysiologic changes that are not fully reversed by mitral repair with widespread adoption of ablation and tricuspid repair.

Several mechanisms may underlie the adverse outcomes observed in AF patients. First, AF reflects chronic LA pressure and volume overload, leading to atrial fibrosis, impaired atrial reservoir function, and increased risk of thromboembolism even after restoration of sinus rhythm. Second, AF is often associated with right-sided heart disease, as demonstrated by the higher prevalence of moderate-severe TR and elevated TR gradients. It was noteworthy to find greater TR severity long-term in AF patients despite a significantly higher prevalence of tricuspid annuloplasty, suggesting that TR aggravation after mitral repair occurred not only from the annular dilation but also from right ventricular remodeling not amenable to surgical correction. This interaction may contribute to persistent symptoms and right heart failure after surgery. Third, our longitudinal echocardiographic analysis revealed that AF patients exhibited attenuated reverse remodeling of both the left ventricle and left atrium, with greater values of estimated pulmonary pressures—indicators of ongoing hemodynamic stress and substrate vulnerability.^10^ This structural substrate may explain the higher rates of adverse events including heart failure, cerebral infarction, catheter ablation, and pacemaker implantation despite initial surgical ablation and careful anticoagulation and rhythm surveillance even after LA appendage closure and ablation.

### Clinical Implications

Our present results have several important clinical implications. First, our data demonstrate that AF in patients with degenerative MR undergoing MV repair is a robust marker of adverse long-term prognosis, even in the setting of frequent concomitant surgical ablation and tricuspid repair. When patients with baseline AF were stratified according to AF type (ie, paroxysmal vs persistent), those with persistent AF exhibited larger LA size, a higher prevalence of TR severity ≥moderate, and lower freedom from composite adverse events compared to patients with paroxysmal AF (Figure 5, Figure E2). These findings suggest that atrial remodeling already may be advanced at the time of surgery in many patients with persistent AF.^18^ From a clinical perspective, earlier referral for MV repair—before the development of sustained AF or marked atrial enlargement—may help preserve atrial structure and function, improve the durability of sinus rhythm, and ultimately translate into better long-term outcomes. These observations support current guideline recommendations advocating timely intervention for MR and emphasize the importance of incorporating atrial rhythm status into surgical decision making.^5^

Second, recent refinements in surgical ablation techniques across different surgical eras also may influence long-term clinical and rhythm outcomes. When the study period was divided into 3 eras—2001-2011, 2012-2018, and 2019-2022—the prevalence of surgical ablation among patients with atrial fibrillation did not differ significantly across eras (91.4%, 93.4%, and 89.5%, respectively; *P* = .624). However, left-sided ablation strategies were adopted more frequently in more recent eras (3.5% vs 10.6% vs 13.8%; *P* < .001). In contrast, the use of LA appendage occlusion increased markedly over time (32.3% vs 65.9% vs 84.8%; *P* < .001). With respect to the evolution of ablation strategy, ablation of the coronary sinus through the oblique sinus has been routinely incorporated since 2019. In parallel, advances in LA appendage exclusion—particularly the adoption of epicardial clip devices—have been reported to improve the reliability and completeness of appendage closure, potentially reducing residual thromboembolic risk.^19^ Indeed, in the most recent era, LA appendage occlusion was performed predominantly using epicardial clip devices (91%). Despite these technical advances, the 5-year cumulative incidence of cerebral infarction in the overall cohort did not improve over the study period (3.1% in 2001-2011, 2.5% in 2012-2018, and 4.5% in 2019-2022; *P* = .439). Moreover, even in the most recent era characterized by aggressive LA appendage occlusion strategies (2019-2022), preoperative AF remained associated with a significantly higher risk of cerebral infarction compared with sinus rhythm (5-year cumulative incidence, 9.0% vs 2.7%; *P* = .012).

Taken together, these findings suggest that even with contemporary ablation techniques and LA appendage management strategies, preoperative AF remains an important prognostic marker, likely reflecting advanced or irreversible atrial remodeling present at the time of surgery. They also indicate that technical refinements alone may be insufficient to fully offset advanced atrial disease and underscore both the value and the limitations of guideline-recommended concomitant AF surgery, reinforcing the need for careful patient selection based on such factors as LA size, AF chronicity, and comorbidity burden.^11^^,^^12^^,^^16^^,^^17^

Importantly, our data do not establish a causal relationship between surgical timing and outcomes; rather, they indicate that once AF is present, long-term risks remain elevated despite contemporary surgical strategies. In this context, the development of AF should be recognized as a clinically meaningful marker of advanced atrial disease that warrants careful reassessment of disease stage, rhythm management expectations, and long-term risk even after successful MV repair rather than being viewed solely as a factor modifiable by surgical timing. Future prospective studies are needed to determine whether intervention before the onset of advanced atrial remodeling—or before the development of AF—can modify long-term outcomes and promote atrial reverse remodeling following MV repair.

### Limitations

This was an observational study conducted at a single institution, which may limit its generalizability, despite the large sample size and long-term follow-up. Although postoperative rhythm status was assessed at several time points, episodes of postoperative asymptomatic AF might have been missed, because electrocardiographic recordings and Holter monitoring were performed primarily during scheduled outpatient visits or when symptoms occurred. Therefore, the precise timing and frequency of transitions between sinus rhythm and AF could not be determined, limiting our ability to assess the impact of postoperative rhythm transitions on adverse events. The surgical era spans 2001 to 2022, during which time changing practices (eg, minimally invasive/robotic approaches introduced in 2011 and after 2018) may have potentially introduced temporal procedural bias.

**Figure E3:**
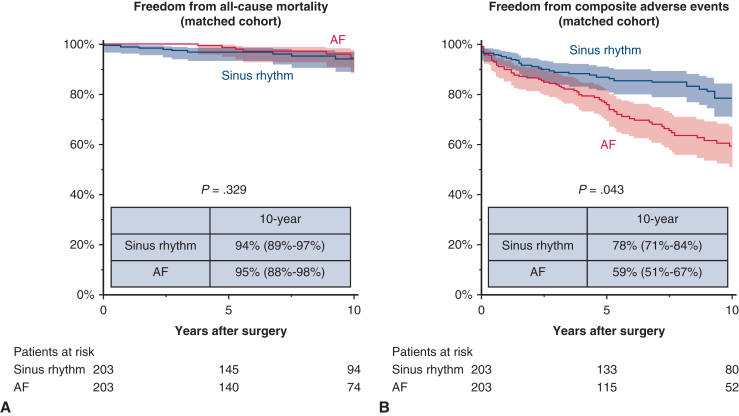
Kaplan-Meier estimates of freedom from all-cause mortality (A) and composite adverse events (B) in a propensity score–matched cohort (n = 203 per group) in which left atrial LA dimension was included in the matching model. Shaded areas represent 95% confidence intervals, and numbers at risk are shown below each panel. *AF*, Atrial fibrillation.

Although covariate balance after propensity score matching was assessed using SMD, a relatively lenient threshold (SMD <0.25) was applied to preserve sample size and covariate overlap in this heterogeneous cohort. As a result, modest residual imbalance may remain and should be considered when interpreting the findings. Furthermore, unmeasured confounders may have influenced the outcomes. Particularly, LA dimension is not only a consequence of AF but also a cause of AF, suggesting that A remodeled LA is more likely to produce ectopic/aberrant rhythms, and this LA dimension could be a confounder. To address this concern, we incorporated LA dimension as an additional covariate into the propensity score model, along with the 15 preoperative variables and reanalyzed the matched cohorts. Consequently, the principal findings—particularly comparable long-term survival and lower freedom from composite adverse events for patients with AF—remained unchanged (Figure E3).

**Figure E4:**
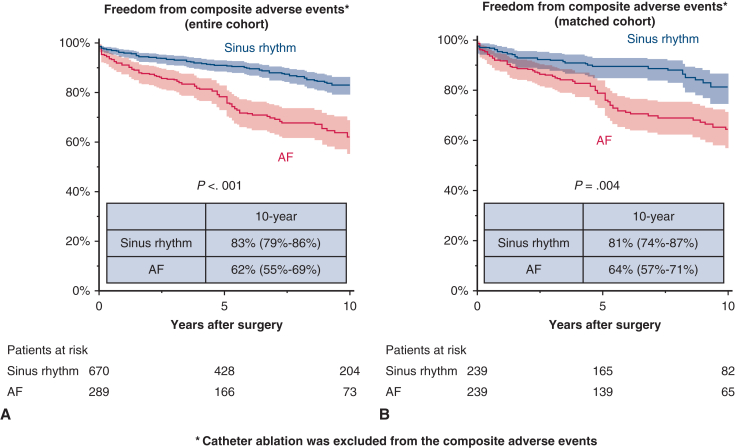
Kaplan-Meier estimates of freedom from composite adverse events in the unmatched (A) and propensity score–matched (B) cohorts. Shaded areas represent 95% confidence intervals, and numbers at risk are shown below each panel. *AF*, Atrial fibrillation.

Because the primary endpoint was analyzed as time to first event and included all-cause mortality as a component, competing risks were not modeled separately. As a result, the composite outcome reflects the timing of the first event rather than the cumulative incidence of individual nonfatal outcomes, and differential mortality between groups may have influenced the observed distribution of endpoint components. Moreover, catheter ablation may be influenced by baseline rhythm status and by management strategy, and its inclusion in the composite endpoint could introduce ascertainment and treatment-related bias. Therefore, we performed a sensitivity analysis excluding catheter ablation from the primary composite endpoint and repeated the long-term time-to-event analyses in the overall cohort and propensity score–matched cohort. The association between preoperative AF and adverse outcomes remained consistent (Table E3, Figure E4).

## Conclusions

Among patients undergoing MV repair for degenerative MR, preexisting AF was independently associated with worse long-term outcomes and attenuated LA reverse remodeling, despite aggressive concomitant surgical interventions. Therefore, AF should be recognized as a marker of advanced atrial disease and increased postoperative risk, highlighting the need for individualized, guideline-informed decision making and continued investigation into strategies that may improve outcomes in this high-risk population.

## Conflict of Interest Statement

The authors reported no conflicts of interest.

The *Journal* policy requires editors and reviewers to disclose conflicts of interest and to decline handling or reviewing manuscripts for which they may have a conflict of interest. The editors and reviewers of this article have no conflicts of interest.

## Figures and Tables

**Table E1:** Determinants of composite adverse events in the entire cohort

Variable	Univariate		Multivariate	
	*P* value	HR (95% CI)	*P* value	HR (95% CI)
Clinical variables				
Age (per 10 y)	<.001	1.5 (1.4-1.7)		
Age older than 60 y	<.001	2.3 (1.8-3.1)	<.001	1.9 (1.4-2.6)
Male	.830			
Body surface area	.004	0.4 (0.2-0.7)	.233	
Symptomatic	.010	1.9 (1.2-2.9)	.081	
Atrial fibrillation	<.001	2.1 (1.6-2.7)	.010	1.5 (1.1-2.0)
Hypertension	.334			
Hyperlipidemia	.402			
Diabetes	.912			
COPD	.025	1.5 (1.1-2.0)	.490	
Chronic kidney disease	.180			
Creatinine	.011	1.3 (1.1-1.5)	.035	1.2 (1.0-1.4)
Echocardiographic data				
LVESD	.591			
LVESD >40 mm	.473		.811	
LVEF	.144			
LVEF <60%	.047	1.3 (1.0-1.7)	.128	
LA dimension, mm	<.001	1.03 (1.01-1.04)	.146	
TR grade >moderate	.003	1.8 (1.2-2.5)	.914	
Leaflet involvement				
Posterior only	.005	0.7 (0.5-0.9)	.005	0.7 (0.5-0.9)
Anterior or bileaflet	1.000			

*HR*, Hazard ratio; *CI*, confidence interval; *COPD*, chronic obstructive pulmonary disease; *LVESD*, left ventricular end-systolic dimension; *LVEF*, left ventricular ejection fraction, *LA*, left atrial; *TR*, tricuspid regurgitation.

**Table E2:** Determinants of composite adverse events in the matched cohort

Variable	Univariate		Multivariate	
	*P* value	HR (95% CI)	*P* value	HR (95% CI)
Clinical variables				
Age (per 10 y)	<.001	1.5 (1.3-1.8)		
Age older than 60 y	<.001	2.2 (1.5-3.4)	<.001	2.3 (1.5-3.4)
Male	.905			
Body surface area	.061			
Symptomatic	.198		.183	
Atrial fibrillation	.001	1.8 (1.3-2.5)	<.001	1.8 (1.3-2.5)
Hypertension	.288			
Hyperlipidemia	.535			
Diabetes	.157			
COPD	.168			
Chronic kidney disease	.048	2.0 (1.0-3.7)	.016	2.2 (1.1-4.2)
Creatinine				
Echocardiographic data	.076			
LVESD	.441			
LVESD >40 mm	.354		.817	
LVEF	.529			
LVEF <60%	.145		.171	
LA dimension, mm	.053			
TR grade> moderate	.082			
Leaflet involvement				
Posterior only	.054	0.7 (0.5-1.0)		
Anterior or bileaflet	1.000			

*HR*, Hazard ratio; *CI*, confidence interval; *COPD*, chronic obstructive pulmonary disease; *LVESD*, left ventricular end-systolic dimension; *LVEF*, left ventricular ejection fraction; *LA*, left atrial; *TR*, tricuspid regurgitation.

**Table E3:** Determinants of composite adverse events in the entire cohort

Variable	Univariate		Multivariate	
	*P* value	HR (95% CI)	*P* value	HR (95% CI)
Clinical variables				
Age (per 10 y)	<.001	1.6 (1.4-1.9)		
Age >60 y	<.001	2.5 (1.9-3.5)	<.001	2.0 (1.5-2.9)
Male sex	.937			
Body surface area	.002	0.3 (0.2-0.7)	.191	
Symptomatic	.003	1.7 (1.2-2.6)	.084	
Atrial fibrillation	<.001	2.1 (1.6-2.7)	.033	1.4 (1.0-1.9)
Hypertension	.060			
Hyperlipidemia	.178			
Diabetes	.544			
COPD	.081			
Chronic kidney disease	.180			
Creatinine	.013	1.3 (1.1-1.5)	.016	1.2 (1.0-1.5)
Echocardiographic data				
LVESD	.446			
LVESD >40 mm	.736		.520	
LVEF	.170			
LVEF <60%	.120		.148	
LA dimension, mm	<.001	1.03 (1.01-1.05)	.039	1.02 (1.00-1.04)
TR grade > moderate	<.001	1.6 (1.2-2.2)	.928	
Leaflet involvement				
Posterior only	.160			
Anterior or bileaflet	1.000			

*HR*, Hazard ratio; *CI*, confidence interval; *COPD*, chronic obstructive pulmonary disease; *LVESD*, left ventricular end-systolic dimension; *LVEF*, left ventricular ejection fraction; *LA*, left atrial; *TR*, tricuspid regurgitation.
